# Comparison of 432 *Pseudomonas* strains through integration of genomic, functional, metabolic and expression data

**DOI:** 10.1038/srep38699

**Published:** 2016-12-06

**Authors:** Jasper J. Koehorst, Jesse C. J. van Dam, Ruben G. A. van Heck, Edoardo Saccenti, Vitor A. P. Martins dos Santos, Maria Suarez-Diez, Peter J. Schaap

**Affiliations:** 1 Wageningen University, Laboratory of Systems and Synthetic Biology, Wageningen, 6708 WE, The Netherlands; 2 LifeGlimmer GmbH, Berlin, Germany

## Abstract

*Pseudomonas* is a highly versatile genus containing species that can be harmful to humans and plants while others are widely used for bioengineering and bioremediation. We analysed 432 sequenced *Pseudomonas* strains by integrating results from a large scale functional comparison using protein domains with data from six metabolic models, nearly a thousand transcriptome measurements and four large scale transposon mutagenesis experiments. Through heterogeneous data integration we linked gene essentiality, persistence and expression variability. The pan-genome of *Pseudomonas* is closed indicating a limited role of horizontal gene transfer in the evolutionary history of this genus. A large fraction of essential genes are highly persistent, still non essential genes represent a considerable fraction of the core-genome. Our results emphasize the power of integrating large scale comparative functional genomics with heterogeneous data for exploring bacterial diversity and versatility.

The *Pseudomonas* genus exhibits a broad spectrum of traits and *Pseudomonas* species show a remarkable adaptability to the biochemical nature of the large variety of environments, often extreme, they thrive in^1^,^2^. The genus currently includes almost 200 recognized species, which have been clustered into seven groups and into lineages on the basis of a limited set of loci^3^. Some species are well-studied because they are human or plant pathogens, like *P. aeruginosa* or *P. syringae*, or because they are considered harmless and possess interesting biodegradation properties while others can produce a variety of extraordinary secondary metabolites with anti-microbial properties^4^. *P. putida* KT2440 is even Generally Recognized as Safe (GRAS-certified) for expression of heterologous genes and has been transformed into a genetically accessible laboratory and industrial workhorse^5^.

A number of comparative genomics studies have been performed in the past^1^,^3^,^6^ but the number of available *Pseudomonas* genomes quadrupled in the last five years due to the widespread use and the advancement of high-throughput sequencing technologies. As of December 2015, the complete and draft genomes of 432 strains distributed over 33 species are publicly available (see Supplementary Figure S1). This plethora of data entitles an in-depth comparative re-analysis of *Pseudomonas* genomes to explore their metabolic and ecological diversity.

Large scale functional comparison based on sequence similarity is challenged by methodological problems, such as the need of of defining arbitrarily generalized minimal alignment length and similarity cut-off for all sequence to be analyzed, and it is hampered by the high computational cost, since time and memory requirements scale quadratically with the number of genome sequences to be compared^7^. Many bacterial proteins consist of two or more domains and fusion/fission events are the major drivers of modular evolution of multi-domain bacterial proteins^8^. Interspecies domain variation can thus give rise to an annotation transfer problem: sequence based functional annotation methods use a consecutive alignment to identify common ancestry and therefore may miss domain insertion/deletion, exchange or repetition events, which may lead to functional shifts and promiscuity. Comparisons at protein sequence level should therefore be complemented with comparisons at the protein domain level^7^. In addition, in order to avoid technical biasses a biologically meaningful functional comparison requires consistent and up-to-date annotations. Instead, the biological information available in public databases varies in quality due to the use of different databases and annotation pipelines that include different methods and may assign different names, acronyms and aliases to the same protein. Re-interpretation of these predictions in most cases requires reverse engineering as data provenance is usually not available.

In this paper 432 *Pseudomonas* genome sequences were *de novo* re-annotated and the generated annotation information was integrated through a semantic platform with data from six metabolic models, nearly a thousand transcriptome measurements and four large scale transposon mutagenesis experiments. We identified phylogenetic relationships among different species using protein domains and performed extensive analysis of the core- and pan-genomes of the *Pseudomonas* genus and considered the habitat factor while analyzing the pan/core-genome. Finally, we linked domain content and domain variability of persistent and essential genes and their transcriptional regulation.

## Results

### *De novo* annotation of *P. putida* KT2440 as a minimal working example

**Figure 1:**
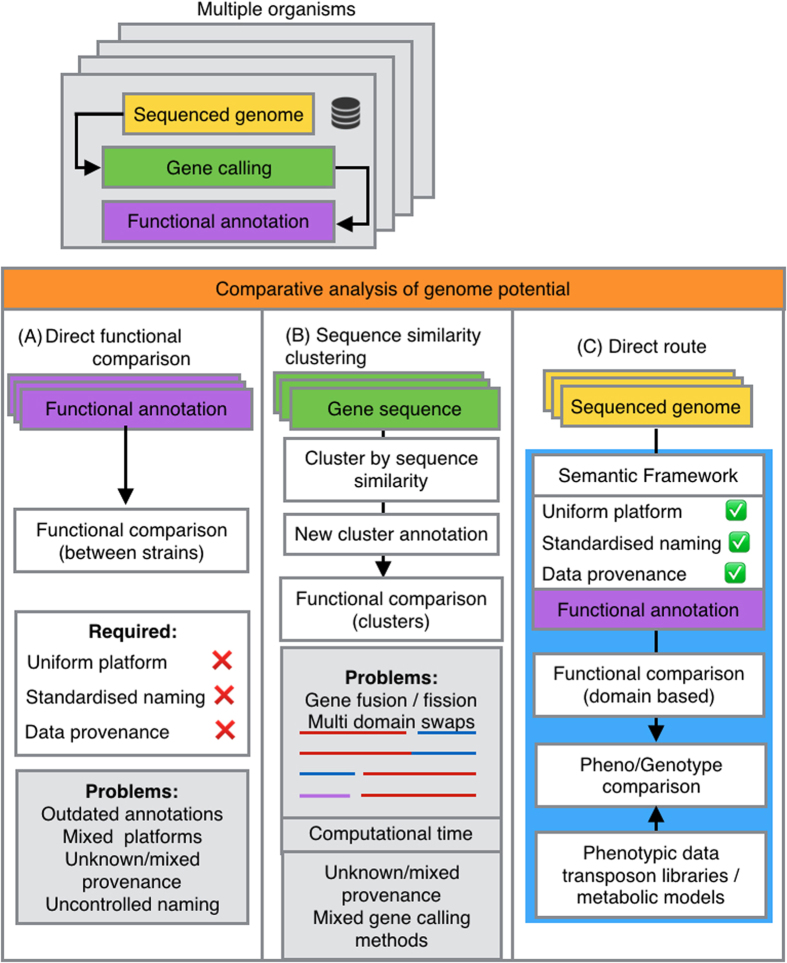
Alternatives for functional genome comparison. (**A**) Direct comparison of genome potential using existing annotation is often hampered by lack of standardization of gene calling and annotation tools, mixed and unknown data provenance and inconsistent naming of function. (**B**) Sequence similarity clustering bypasses inconsistent functional annotations. Computational time scales quadratically with the number of genome sequences and gene fusion/fission events might be overlooked. (**C**) Usage of standardised annotation tools ensures uniform genome annotation prior to comparison; annotation provenance is stored for all steps.

*P. putida* KT2440^5^ is one of the best-characterized *Pseudomonas* strains. A *de novo* annotation obtained using an in-house annotation pipeline, the annotation deposited in GenBank (NC_002947) and an alternative annotation obtained using RAST^9^ were compared, see Table 1. The total number of genes identified using three gene calling methods, Prodigal 2.6 (in our pipeline), Glimmer3 (RAST), and Glimmer (GenBank) are very similar, differing less than 4%. However, as each of these algorithms have an intrinsic false discovery rate in start-site prediction, significant differences in the start position of the identified genes were found. The number of exact matches in gene start-sites is only 73% (4073 genes) confirming previous observations^10^. These 5′ variations in gene identification can result in a putative gain or loss of biological functions; however, since different naming conventions are used in the different annotation protocols applied, a direct functional comparison to spot possible differences is not possible (Fig. 1).

The use of controlled vocabularies overcomes this issue, so that functional comparison can be performed using gene ontology (GO) terms, Enzyme Commission (EC) numbers and InterPro identifiers. For the GenBank deposited annotation no GO information was available but the difference observed between the RAST and the *de novo* annotation is striking. This minimal working example shows that even for a single genome a comparative analysis of functional annotations derived from three work-flows is almost impossible by computational means due to lack of standardization and data provenance. This example further emphasizes that comparative genomic analysis requires homogeneous annotation.

### Comparison of the genomic potential of *Pseudomonas* species

Since for a comparative genomics study a consistent and standardized genome annotation is a prerequisite, we evaluated the impact by comparing the functional annotations of 432 *Pseudomonas* genomes with a *de novo* annotation. We used both complete and draft genomes. According to the quality metric defined by Cook and Ussery, almost 30% of the available draft genomes were of low quality^11^. This was mostly due to a high number of contigs and not to the quality of the assemblies in itself, so they were included in the analysis.

**Figure 2:**
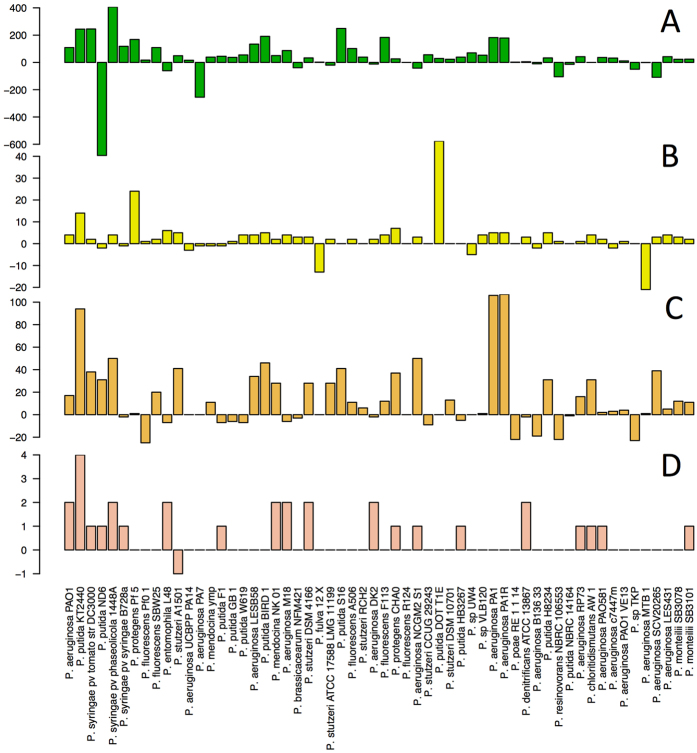
*De novo* annotation of *Pseudomonas* genomes. Comparison between the original and *de novo* annotations of 58 completely sequenced *Pseudomonas* genome sequences. Barplots indicate differences in the number of retrieved genome features terms between the *de novo* annotations and the original deposited annotations. (**A**) gene abundance; (**B**) protein domains; (**C**) GO terms, and (**D**) EC identifiers. The genomes are ordered from left to right by deposition date in the NCBI database (from oldest to newest).

GenBank files were converted into RDF, extracting genome sequences and gene-calls. Genomes were structurally and functionally re-annotated. The originally deposited gene-calls were functionally re-annotated as well and a pairwise comparison of GO terms, and EC identifiers assigned to the originally deposited and the *de novo* gene-calls was performed at gene and protein domain level. Figure 2 summarizes the results for the available 58 complete genomes. Differences in annotations were observed at all functional levels. Per genome on average 38 new genes were predicted while a functional re-annotation of the set of complete genomes yielded 838 additional GO-terms and 146 additional domains (For a more detailed overview see Supplementary Data S2). Considering the full set of 432 genomes, on average a difference of 153 genes per genome was detected. The results advocate for routine implementation of consistent gene-calling methods combined with an up-to-date functional annotation before performing comparative genomic analyses, as many of these differences will results in gain or loss of biological functions.

### Sequence and function based comparative genomics of *Pseudomonas*

Genome-wide comparative analysis usually relies on sequence similarity clustering based on a blast-based all-against-all bidirectional best hit (BBH) heuristic approach. There are several limitations to this approach. Firstly, the runtime increases quadratically with the number and complexity of the species involved. Secondly, clustering is strongly context-dependent as it dramatically depends on chosen cut-off values to define statistical significance of sequence similarity. Problems may arise with in-paralogous sequences that evolve at very similar rates resulting from recent duplication events^12^. Thirdly, protein fusion and fission events are difficult to detect using alignments and thus critical information might be lost.

**Figure 3:**
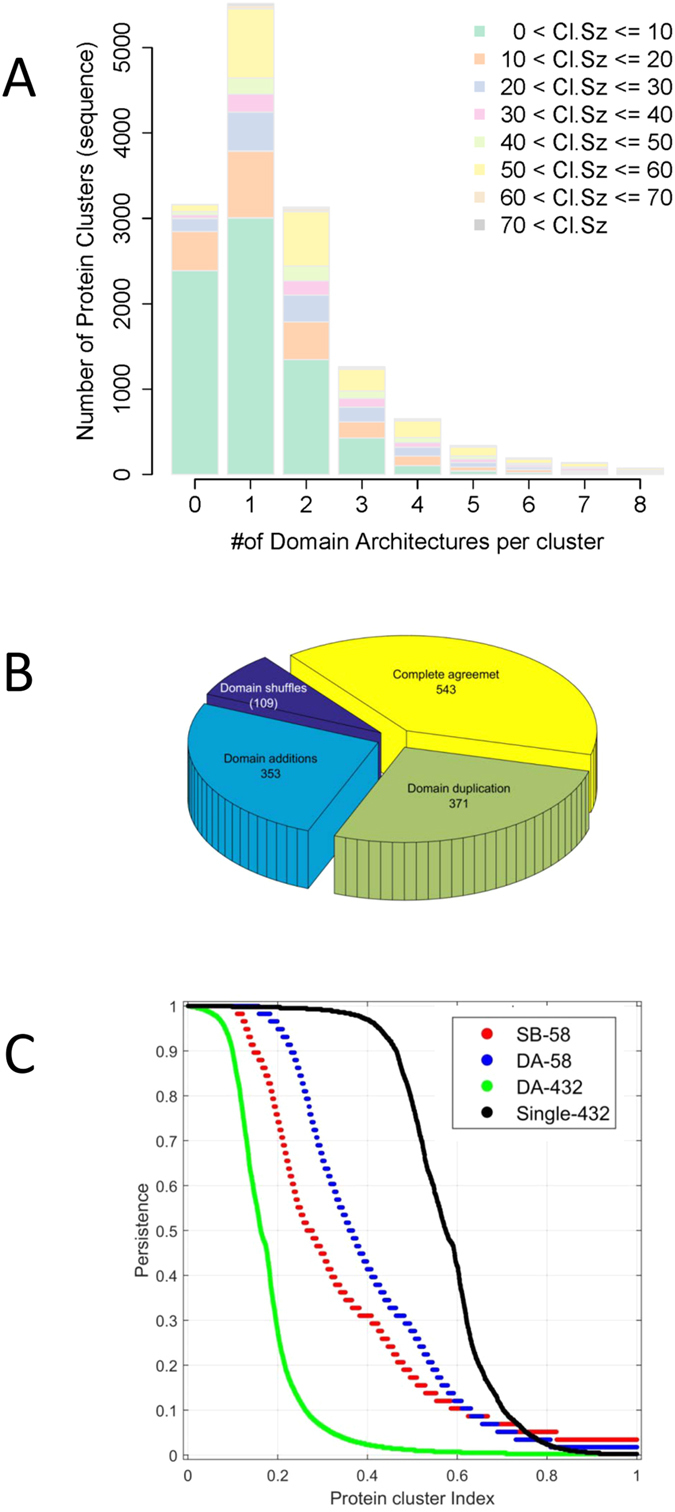
Domain architectures in sequence based clusters of orthologous proteins. (**A**) Number of distinct domain architectures per cluster (**B**) Variability in domain architectures per gene cluster in core-genome. Complete agreement indicates a unique domain architecture shared by all members of the cluster; For the cases where multiple domain architectures were found in a sequence cluster, the number of cases corresponding to domain duplications, additions and shuffles are indicated. (For A and B only 58 complete genome sequences considered). (**C**) Persistence analysis within the Pseudomonas genus. The curves indicate the persistence of each of the cluster. Clusters have been arranged by decreasing persistence values and the *x*-axis has been scaled to 0–1 range, in this way the cluster with the highest persistence have an *x* value of 0 and the cluster with the lowest persistence has an *x* value of 1. The *y*-axis indicates the persistence of a given cluster (see Equation 1): for instance a persistence of 0.8 indicates that 80% of the analyzed genomes contain sequences in that given cluster. SB-58 refers to the use of sequence based cluster considering the 58 complete genomes; DA-58 and DA-432 refers to the use of protein domains, for 58 and 432 genomes respectively; Single-432 reproduces the analysis for single domain proteins found in the full set 432 genome sequences.

An alternative approach, already employed in a comparative genomics study of *Escherichia coli*^13^, consists of grouping of proteins on the base of domain architectures with a fixed N-C terminal order^14^. Clustering based on domain order is highly scalable and moreover, most protein domains represent structural folds that can be directly linked to function. Here, both approaches were compared. Protein sequence similarity clusters were identified in a BBH approach using orthAgogue^15^. Due to runtime constraints, protein clustering was limited to the analysis of the 58 complete genomes leading to the identification of 14757 protein clusters. For each protein found within a cluster the domain content and N-C terminal domain order ranked by the position of the first detected amino acid of the domain (domain start) in the protein sequence (domain architectures) was analysed and is summarized in Fig. 3A. 5515 sequence based protein clusters (37%) present a one-to-one correspondence to domain architectures, whereas 3134 (21%) can be associated to two distinct domain architectures. Overall, 93% of the identified clusters can be associated to 4 or less distinct domain architectures. Figure 3A also shows the number of proteins in each orthologous cluster. 3162 clusters (21%) contain proteins lacking established domains and almost 75% of them contain less than 10 sequences. These clusters correspond, in their vast majority, to hypothetical proteins. Regarding the core genome, 1618 clusters (11%) were found to be present in all 58 genomes. From these 1618 protein clusters, 242 contained duplication events leaving 1376 distinct single copy gene protein clusters common to all 58 genomes. 543 of those clusters showed a single domain architecture whereas the rest contained domain architecture variations as summarized in Fig. 3B. We noted that such variability was mainly due to swapping or inversion in domains order. In a sequence based approach domain order variation can potentially lead to false negatives, broken clusters and even reduction of the core genome when more genomes are added to the analysis.

The analysis of 58 complete genome sequences showed that domain architectures retain enough information for functional characterization and that they can be used as a fingerprint for a functional cluster. Since the computational cost for obtaining protein domain identification scales linearly with number of genomes and can be easily distributed over multiple machines, we used these functional fingerprints to extend the analysis to all 432 *Pseudomonas* genomes. Over two million (2,704,339) genes were identified coding for over one million (1,196,884) unique protein sequences of which 85.6% (1,024,877) contain known protein domains. Figure 3C shows the results of persistence analysis, reporting the fraction of the total number of analysed genomes in which the corresponding cluster/protein domain/domain architecture was found; 40% of the protein domains are persistent in the genus, showing that the functional information at domain level is preserved.

### Classification of *Pseudomonas* strains based on genome potential

**Figure 4:**
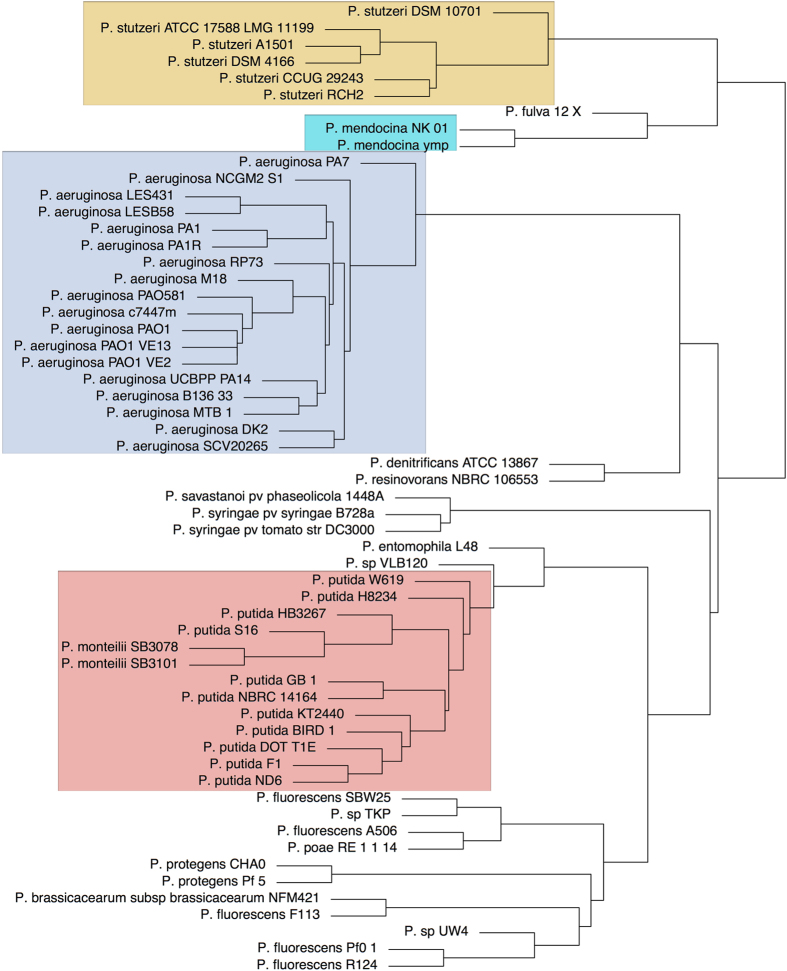
Domain based distance tree of 58 *Pseudomonas* strains. The tree was build considering the pattern of presence/absence of protein domains using an average clustering approach. Only completely sequenced genomes are considered. The phylogenetic clusters corresponding to the most abundant species (*P. stutzeri*, *P. mendocina*, *P. aeruginosa and P. putida*) are colour-shadowed.

Patterns of protein domain presence/absence can provide an alternative and complementary way for assessing strain diversity^16^,^17^. There are still many unclassified *Pseudomonas* strains and there is a continuous development on assessing the phylogeny using various approaches^18^. Figure 4 shows a distance tree of genome potential based on presence/absence of protein domains for the 58 complete *Pseudomonas* genomes. We found excellent agreement between this distance tree and the taxonomic classification based on 16 S sequences indicating that binary patterns of protein domains retain enough information to reconstruct evolutionary history. The positioning of *Pseudomonas* sp. UW4 within the clade of *P. fluorescence*, confirms a previous observation based on 16 S and three housekeeping genes (*gyrB*, *rpoB* and *rpoD*)^19^. *P. aeruginosa* and *P. stutzeri* clades are conserved while *P. putida* and *P. fluorescence* clades shows the addition of different species.

We further extended the domain based distance analysis to include all 432 *Pseudomonas* strains (see Supplementary Figure S3). The majority of the strains cluster in accord with their taxonomic classification. Many of the unclassified strains could be classified either in *P. aeruginosa* (4) or *P. putida* (13).

### Exploring the pan- and core-genome of *Pseudomonas* at protein domain level

The core-genome of a taxon level is defined as the genes persistently present in the population, while the pan-genome is essentially the amount of different genes found within a population at the specified taxonomic level^20^. The currently available genomes allow to measure the pan- and core-genome sizes, however these sizes change upon the addition of new sequences. The core-genome is usually reduced and the pan-genome increases mostly due to the discovery of novel accessory genes that accumulate by lateral transfer, forming new trait combinations until saturation has been reached. Saturated pan-genomes with a stable core-genome are called closed. From the currently available genomes an estimation can be made, using mathematical modelling^20^, of the size of the pan- and core- genomes that are expected if the sequences of every existing strain were to be included in the analysis. We refer to these estimations as estimated pan- and core- genome sizes.

**Figure 5:**
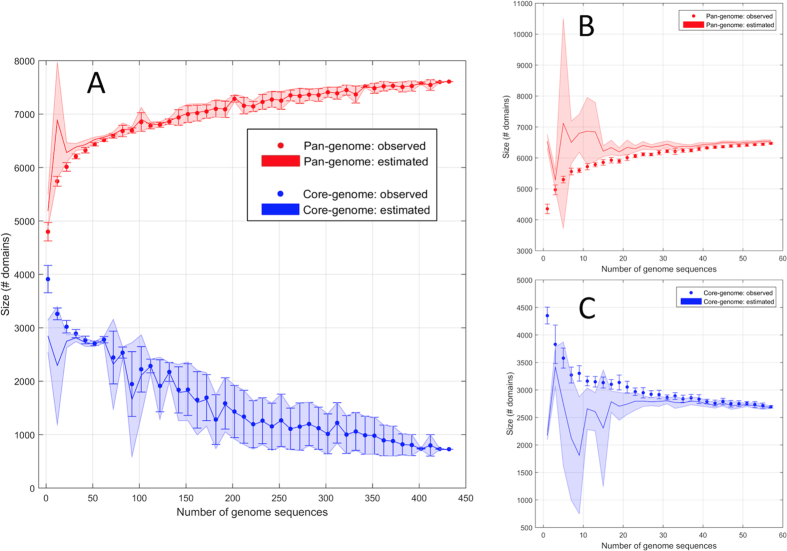
*Pseudomonas* pan- and core-genome defined on the base of protein domains. (**A**) Complete overview of the distribution of the size of the pan- and core- distribution of protein domains. Error bars correspond to standard deviations based on 10 measured random realizations of the indicated number of genomes whereas the shadowed area is the estimated standard deviation using the same approach. (**B**) Pan-genome of the 58 fully circular genomes. (**C**) Core-genome of the 58 fully circular genomes.

Genome potential of the genus *Pseudomonas* is reflected in its metabolic diversity which allows individual species to inhabit a wide variety of environments. With the current set of 432 (draft) genomes we studied whether the observed diversity in genome potential reflects a closed pan-genome. We initially considered the 58 complete genomes. Observed core-genome of 2687 protein domains was to be confronted with an estimated size of 2681. For the pan-genome we found 6472 protein domains (observed) versus 6541 (estimated). Since these measures depend on the number of genomes considered, we explored how these measures vary by using a different number of genomes (from 5 to 58). This was achieved by applying a 10-fold random re-sampling from the 58 genomes to obtain an indication of the possible variability (Fig. 5). As expected the size of the core-genome of the genus decreases with the number of genomes considered while that of the pan-genome increases. The observed and estimated sizes of both the pan- and core-genome are rather stable with respect to the number of genomes used in the calculation, except for small sample size (<15).

Including draft genomes in the calculations resulted in a dramatic reduction, up to the 73%, of the size of the core-genome both observed and estimated, which dropped to 726 and 720 protein domains architectures, respectively. Interestingly, this reduction does not lead to a loss of functional information since single domains are highly persistent as previously stated (40%).

We observed a large variability for both measures. The reduction of the core size and its variability can be partly explained due to the inclusion of draft genomes with a high number of gaps containing non-sequenced genes. The difference between observed and estimated sizes reduced to only one protein domain for both the pan- and core-genome, indicating saturation. Addition of new genome sequences to the analysis will most likely not lead to the identification of a significant set of new domains within this genus. This saturation effect does not depend on the particular estimation model used. Saturation of the pan-genome was also seen through a heap model (*α* = 1.30 ± 0.05). In this analysis values > 1 indicate a closed pan-genome^21^.

### Essentiality analysis of domains in the core-genome

From a functional point of view, the core-genome of a genus is most likely enriched in essential genes necessary for (long term) viability and adaptation to ever changing environmental conditions. Since persistence can be used to identify genes required for survival^22^,^23^, a positive correlation between persistence (the number of genomes sharing a given gene) and essentiality can be hypothesized. To verify this hypothesis we combined gene essentiality measures with gene persistence in the genus. Gene essentiality was defined from experimental results available for two *P. aeruginosa* strains (PAO1 and PA14)^24^,^25^ and from *in silico* predictions. For the latter, we considered 6 genome-scale constraint-based metabolic models which rely on functional annotation to uncover the metabolic potential of biological systems and are able to accurately predict gene essentiality in a large variety of growth conditions^26^.

**Figure 6:**
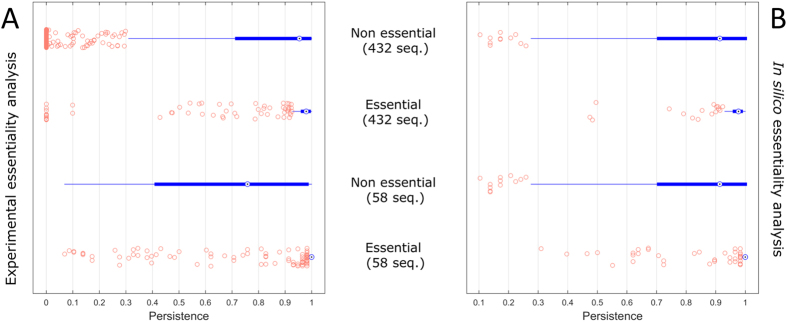
Persistence of (non) essential genes. (**A**) Persistence of essential and non-essential genes as derived by experimental investigations. (**B**) Persistence of essential and non-essential genes as derived by *in silico* modelling using genome based constrained metabolic modelling. Results shown pertain the use of the iMO1086 model for *P. aeruginosa* PAO1. In both cases persistence is calculated using the 58 completely sequenced *Pseudomonas* genomes and the complete set of 432 genomes sequences. Magenta (circle) dots indicate outliers.

We observed that essential genes show higher persistence values than non essential ones: this relationship is conserved when persistence is computed either using a sequence similarity based approach on 58 completely sequenced genomes or for 432 genomes by using a domain architecture approach as shown in Fig. 6A.

A comparison of gene persistence and essentiality for the two strains showed that 65% of genes found to be essential for PA14 growth on LB are also essential for growth of PAO1 on either LB, minimal with pyruvate or sputum agar, but only 39% of genes reported to be essential for PAO1 growth were found to be essential for PA14 (See Supplementary Figure S4). This difference could be due to the smaller set of tested conditions. We used a less stringent cut-off for persistence: 0.95 instead of 1 to allow for non-sequenced genes due to incomplete draft genomes. Therefore, we observed that a small fraction of persistent genes is present in only one of the two strains (0.016% and 0.025% for PA14 and PAO1, corresponding to 75 and 47 genes respectively) which are likely to have been lost through evolution.

Analysis of the complete pan-genome revealed that 1252 single copy genes are persistent. Of these, almost one third (404) were found to be essential *in vivo* under three growth conditions (LB, minimal-pyruvate or sputum agar) for *P. aeruginosa* PAO1 strain^24^. Similar ratios were observed for strain PA14.

1112 unique domains were identified in the 404 essential persistent genes and 1340 unique domains in the non-essential but persistent genes. 203 domains were shared between essential and non-essential persistent genes. Essential genes contain a larger repertoire of unique, single copy domains: 404 essential persistent genes contained, on average, 1.53 single copy domains whereas for non essential persistent genes, the average was 0.82.

*In vivo* essentiallity analysis were limited to four conditions. Using metabolic models a wider range of conditions can be explored albeit the analysis is restricted to metabolic genes. We considered six genome scale constraint based metabolic models describing the metabolism of *P. aeruginosa* PAO1 (models iMO1056^27^ and iMO1086^28^), *P. fluorescens* SBW25 (iSB1139^29^) and *P. putida* KT2440 (iJN746^30^, iJP815^31^, and iJP962^28^).

We explored a wide range of growth conditions with varying carbon, nitrogen, phosphorus and sulphur sources and for each medium composition, gene essentiality predictions were performed using Flux Balance Analysis and are summarized in Table 2. Figure 6B shows results for *P. aeruginosa* model iMO1086, confirming what was observed for experimental data. Of the 750 essential metabolic genes that were identified under 3366 media compositions for iMO1086, 169 genes were identified to be essential under experimental conditions whereas 42 genes were essential but not *in silico* (25%). Average persistence over the 58 complete genomes was 0.96 ± 0.14 for predicted essential genes and 0.85 ± 0.24 for non-essential, which we found to be significant (p-value < 0.01 for a Wilcoxon test). When considering the 432 genomes, we still observed difference in the persistence of predicted essential and non essential genes 0.95 ± 0.12 versus 0.89 ± 0.21, p-value < 0.01). Similar results were also obtained when using essentiality predictions for the other metabolic models.

Using metabolic models to simulate media compositions we identified additional genes that were essential in a number of conditions, retrieving on average 1.47 single copy domains per gene, consistently with what observed for essentiality experiments. We further combined the models’ predictions and we inspected genes predicted to be essential in all the tested conditions. For *P. putida*, the three models showed an overlap of 68 essential genes. Interestingly, these genes contained 2.53 single copy domains on average, underpinning previous results: non-essential genes contain domains that are shared with other genes. This can result in the presence of isozymes or of potentially moonlighting enzymes which can step in for essential functions in the case of deletions or mutations.

### Variability of gene expression and its association to persistence and essentiality in *Pseudomonas*

**Figure 7:**
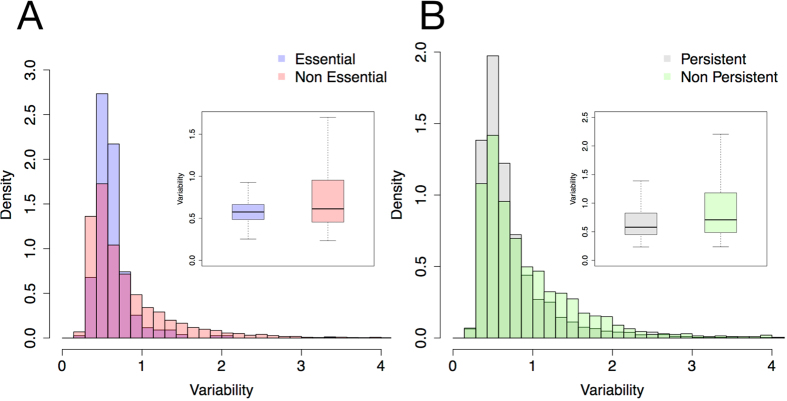
Variability of gene expression levels and its association with persistence and essentiality. (**A**) Distribution of Variability score for (non) persistent genes (genes with persistence lower or higher than 0.95, respectively). Box plots show Variability values for both groups. Difference between mean values is significant (*p*-val < 0.01). (**B**) Distribution of Variability score for essential and non-essential genes with gene essentiality derived experimentally^24^. Box plots show Variability values for both groups. Difference between mean values is significant (*p*-val < 0.01).

Associations between gene essentiality and low variation in protein abundance have been observed in *E. coli*^32^. We hypothesized the existence of an association between gene persistence and expression level variation. We analysed gene expression variability in *P. aeruginosa* using a gene expression compendium containing over 900 samples and 100 datasets regarding *P. aeruginosa* PAO1 genes^33^. Each gene was assigned a score, Variability, for transcriptional variation. Persistent genes tend to show significantly lower degree of variation in expression level than non persistent ones (p-value < 0.01); this holds true also for essential genes (Fig. 7). Similar results are obtained when analysing a more limited dataset containing RNAseq measurements of *P. aeruginosa* PA14 in 14 growth conditions^34^ (see Supplementary Methods S5) This association between low expression variability and persistence/essentiality could indicate that expression of genes in the core-genome is likely to be buffered and independent from environmental growth conditions. To the best of our knowledge such associations have never been established on such large scale due to the limitations associated to comparing hundreds of genome sequences.

## Discussion

For our analysis we did not rely on previously existing annotations, but we performed a consistent re-annotation of all the sequences using a standardized approach that ensured coherence and uniformity. A sequenced based approach was used for a prior comparative analysis to define clusters of orthologous proteins in the smaller dataset of 58 complete genomes. Due to polynomial growth of computational time, this approach is not feasible for large data sets. Mining a gene sequence for domain occurrences is less computationally demanding, which provides an effective scalable approach.

Sequence based approaches are used to identify clusters of orthologous proteins, however the analysis of domain architectures is targeted towards the identification of groups of functionally equivalent proteins. Protein domains provide a standardised way to assess sequence variation and its impact in function, since every amino acid has a characteristic weight in the domain model. Protein domains are more strongly associated to protein structure than protein sequences, thereby providing a closer link to function that can bridge over larger evolutionary distances, which is essential to comparative functional analysis. Still there is a need for improving how protein domain are defined to accommodate similar models arising from, possibly different, databases and to take into account positional variations that might lead to spurious domain inversions.

When applied to the inferred proteomes of the 58 complete genomes, both clustering methods yield similar results. The same clusters were obtained in 40% of the cases meaning that each of these clusters contained an equal number of proteins, captured the same strains and shared the same domain architectures. In 20% of the cases, very similar but numerically distinct clusters were obtained, as a given sequence similarity cluster had captured two distinct domain architectures. In most of these cases variability in domain architecture were caused by changes in domain order due to small variations in the start position of overlapping domains. Approximately, 20% of identified proteins have no recognizable functional domains. As most of these proteins are hypothetical they were not considered for functional analysis. When only proteins containing domains are considered, over 90% of the clusters identified using sequence comparisons contain 4 or less distinct architectures.

The differences in the persistence curves shown in Fig. 3C show that the way the clusters are defined, either using sequence similarity or protein domains, impacts the calculation of gene persistence: this has repercussions on the definition of the core genome and its size. We found these differences to be larger when more genomes are considered. This is more likely linked to the broader range of phylogenetic distances among considered genomes: this is explored in more detail in Koehorst *et al*.^35^.

Our analysis resulted in the identification of the pan- and core-domainome of 432 *Pseudomonas* which is closed according to the heap model as also recently noted for the *P. aeruginosa* species^36^. This suggests that sequencing additional strains will fail to add new genes to the pan-genome: however, this is likely an oversimplification. Here, we understand closeness of the pan-genome as measure of the genus ability to acquire exogenous genes and as a proxy for the ratio between vertical and horizontal gene transfer indicating that horizontal gene transfer has not played a major role in shaping the genome content of the genus.

Key characteristics of *Pseudomonas* must be located in the genus core-genome, however comparison with metabolic models shows that identified core is not autonomously functional. Not all the genes in the core-genome seem to be essential (under given tested conditions), however essential genes represent ≈40% of the core-genome, in agreement with previously reported ratios for other species/genus^37^. The remaining 60% contain unique features defining the genus.

We found a strong association between gene essentiality and protein domain properties. We observe an inverse correlation between the number of proteins in the genome containing the considered domain and essentiality, with average number of domains uniquely present in the considered protein going from 1.5 to 0.8 when non essential/essential genes in the core-genome are considered. The average number of single copy domains per gene further increases when stricter criteria for gene essentiality are applied, namely that genes should be essential in all the simulated media.

Accurate algorithms to predict gene essentiality from genomic features have been also developed and domain enrichment score has been shown to have a high predictive power^38^ which is computed based on the ratio of occurring frequencies of a particular domain between essential genes and the total genes in the whole genome of already characterized species. Here we have established a link between the number of copies of a domain in a genome and gene essentiality that can be used to complement essentiality predictions.

The extensive use of metabolic reconstructions allowed us to identify conditionally essential genes, and a large number of single copy domains is also observed in these genes. This supports the idea that protein domains are the driving force behind gene essentiality which is preserved through protein domains rather than through the conservation of entire genes^39^.

We have shown that lower fluctuations in gene expression are associated to essential and/or persistent genes. Further work is required to clarify the overlap and intertwining between both gene categories (essential/persistent) and to clarify the (possibly different) regulatory mechanisms stabilizing their expression levels.

## Methods

### Genome retrieval

Genbank files containing genome sequences and existing annotations for 58 circular genomes and 374 draft genomes of the *Pseudomonas* genus were downloaded from the GenBank database in June 2015. Annotation of *Pseudomonas* KT2440 was also downloaded from RAST^9^. A detailed list of the included strains is available (see Supplementary Figure S1 and Supplementary Data S2).

### Genome *de novo* annotation

To perform the re-analysis of the 432 gemomes sequences we used a in-house pipeline for annotation and data storage^7^. Likewise existing annotation pipelines such Prokka^40^, it relies on external feature prediction tools to identify the coordinates of genomic features within genomics sequences. The pipeline consists of a number of python modules that execute annotation applications and convert results and provenance directly into the RDF data model with a self defined ontology (the complete description of the implemented ontology can be obtained using RDF2Graph^41^) using the RDFLib library. For genetic elements determination a variety of tools is implemented such as Prodigal^42^ for gene prediction. The main difference is that results are stored as Turtle files^43^ containing an RDF model which allows simultaneous exploration of annotation data of multiple genome sequences, greatly facilitation multiple comparison and the integration of heterogeneous source of information. Since it deploys semantic features allowing the storage of data provenance, we refer to it as SAPP (semantic annotation pipeline with provenance). Annotation can be exported to other formats for downstream processing with other tools such as Roary^44^.

Each genome sequence was converted to the RDF data model using the EMBL/GBK to RDF module. *De novo*. The FASTA2RDF, GeneCaller (a semantic wrapper for Prodigal 2.6^42^) and InterPro (a wrapper for InterProScan^45^) modules were used to handle and annotate the genome sequences. Results were retrieved with SPARQL queries.

### Protein domain presence and phylogenetic analysis

A SPARQL query was used to extract the presence of protein domains for all 432 genomes. Data were stored in a 432 (genomes) by 7608 (protein domains) binary matrix (0/1 for absence/presence). Protein domains were identified by their INTERPRO identifiers. Phylogenetic trees based on protein domains were created taking as input the domain presence/absence matrix. The R package pvclust was implemented in R (version 3.3.1)^46^ with a binary distance and average clustering approach with a bootstrap value of 10^47^.

### Protein domain architecture based clustering

The positions (start and end on the protein sequence) of domains having InterPro^48^ identifiers were used to extract domain architectures (*i.e.* combinations of protein domains). Protein domains were retrieved for each protein individually. The domain starting positions were used to assess relative position in the case of overlapping domains; alphabetic ordering was used in the case of domains with the same starting position. Labels indicating N-C terminal order of identified domains were assigned to each protein so that the same labels were assigned to proteins sharing the same domain architecture. Here we have followed a strict approach and two domain architectures were considered different whenever they had different domains or they appeared in different order. For more details see Koehorst *et al*.^35^.

### Estimation of pan- and core-genome size

The estimated number of domains in the pan- and core-genomes expected if the sequences of every existing strain were to be included in the analysis were computed using binomial mixture models as implemented in the micropan R package^49^ using the domain presence/absence matrix previously defined and default values for the parameters. *Pan-* and *core-* analysis was initially performed on the 87 genomes with a maximum of 3 contigs to avoid bias due to incomplete genome sequences. Analysis was extended to the remaining 374 draft genome sequences available. To obtain an indication of the variability of these measures as function of the number of sequences used, these were calculated by a 10 fold random sampling from the full set. Heap analysis as implemented in the micropan R package was used to estimate openness or closeness of the pan-genome^50^ using 500 genome permutations and repeating the calculation 10 times. Final measure is given as the mean ± standard error.

### Orthologous gene detection

Orthologous genes were calculated initially for the set of 58 completely sequenced genomes. Protein sequences predicted using Prodigal 2.6 were extracted using a SPARQL query and used in a Best Bidirectional Hit approach^51^: using an all-versus-all BLASTP comparison and an E-value threshold of 10^−5^ and a maximum target sequence of 10^5^. OrthAgogue^15^ was used to convert BLAST results into a weighted graph. The MCL^52^ clustering algorithm was applied, using an inflation value of 1.5, on the graph to define protein clusters. The results were then extrapolated to the full set of 432 genomes using cluster specific domain fingerprints. Specifically, the sequence clusters obtained through MCL clustering on the 58 complete genomes were used to define sets of protein domains (each sequence cluster was mapped to a set of domains). The remaining genomes were then looked for any given domain set defined on the 58 genomes to define their presence/absence in the draft genomes.

### Persistence and essentiality analysis

The persistence of a gene can be defined as


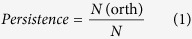


where *N*(*orth*) is the number of genomes carrying a given orthologue and *N* is the number of genomes searched^53^. For the 58 completely sequenced genomes, orthologous genes were inferred using a BBH approach. For the full set of 432 sequenced genomes orthologous genes were inferred by making use of protein domain arrangements.

Locus tags for predicted proteins were inferred from the original annotation through SPARQL. Locus tags were linked to gene essentiality as defined in experimental studies available for *P. aeruginosa* PAO1^24^ and PA14^25^. For each of the predicted proteins with inferred locus tag the corresponding protein cluster was initially calculated for the 58 genomes. The domain architecture corresponding to each cluster was extracted and subsequently scanned against all 432 available sequences. We used the MCL clusters as a reference set for the identification of domain architecture variations which were then extrapolated over the 432 genomes. The persistence for each locus tag was calculated and compared against the essentiality score obtained from two experimental studies.

### Metabolic model essentiality analysis

We considered six genome scale constraint based metabolic models describing the metabolism of *P. putida* KT2440 (models iJN746^30^, iJP815^31^, and iJP962^28^), *P. aeruginosa* PAO1 (models iMO1056^27^ and iMO1086^28^) and *P. fluorescens* SBW25 (model iSB1139^29^). For each genome-scale metabolic model we performed a single gene essentiality analysis in a large number of growth media varying in carbon (C), nitrogen (N), phosphorus (P) and sulphur (S) source. To define the growth media we first identified candidate C, N, P, and S sources in each model independently. Because chemical sum formulas were not always available, we considered each compound for which an exchange reaction was present as a candidate C, N, P and S sources. We changed the *in silico* medium composition to a minimal salts medium containing glucose as C source, ammonia as N source, phosphate as P source, sulphate as S source, in addition to oxygen, water, H^+^, and a variety of salts depending on the particular model considered. The potential of each candidate C, N, P, and S source was then evaluated by adding it to the *in silico* medium while omitting the default C, N, P, or S sources. Growth predictions were performed using Flux Balance Analysis^26^ as implemented in the Matlab COBRA Toolbox^54^. This provided 4 lists of compounds that were suitable as C, N, P or S sources which were then combined into a single list of growth media by taking all combinations of compounds from the 4 lists. For each medium, we then used the *singleGeneDeletion* function from the COBRA toolbox to determine the growth rate of the mutant strains. If a gene knock-out reduced the *in silico* growth rate below 10-6 we considered the gene as essential. Models and Matlab scripts used in this analysis are available in Supplementary Data S6.

### Comparison of gene expression profiles

A publicly available gene expression compendium for *P. aeruginosa* was retrieved^33^. Briefly, this dataset contains a collection of gene expression datasets (950 individual samples pertaining 109 distinct datasets) measured using Affymetrix platform GPL84 and processed using a common normalization and background correction protocol. The final dataset contains expression measurements (in a *log*_2_ scale) for 5549 genes from *P. aeruginosa* PAO1. For every gene we considered its expression profile in this compendium and a Variability value was calculated as the ratio between the standard deviation and the mean.

### Availability of Data and Materials

The annotation pipeline framework is distributed under the MIT license. The pipeline all genomic data, data provenance and computational results associated with this study are freely available at http://semantics.systemsbiology.nl. Additionally, the data associated to this study are provided in turtle format as an RDF serialized dump. This dataset is made available under the Open Database License: http://opendatacommons.org/licenses/odbl/1.0/.

## Additional Information

**How to cite this article**: Koehorst, J. J. *et al*. Comparison of 432 *Pseudomonas* strains through integration of genomic, functional, metabolic and expression data. *Sci. Rep.* **6**, 38699; doi: 10.1038/srep38699 (2016).

**Publisher's note:** Springer Nature remains neutral with regard to jurisdictional claims in published maps and institutional affiliations.

## Supplementary Material

Suplementary Data S6

Supplementary Information

## Figures and Tables

**Table 1:** Annotation results for *P. putida KT2440*.

	#Genes	#Unique start/end positions	#Unique GO	Unique domains	Unique EC
GenBank	5350	170	0	3574	443
RAST	5531	62	726	3631	447
SAPP	5555	252	1403	3636	447

GenBank refers to the original deposited annotation (available at NCBI), whereas RAST and SAPP refer respectively to their annotation.

**Table 2:** Conditional gene essentiality predictions using six metabolic models from three *Pseudomonas* species.

Organism	P. aeruginosa		P. putida			P. fluorescens
Model	iMO1056	iMO1086	iJN746	iJP815	iJP962	iSB1139
*Medium sources*						
#Carbon	49	51	60	40	43	44
#Nitrogen	32	33	22	25	27	19
#Sulfur	4	1	10	1	1	6
#Phosphor	2	2	1	1	1	2
*Genes*						
#Essential/persistent*	115/106	149/132	118/104	112/100	162/148	117/95
#Conditional/persistent*	591/278	601/278	389/170	113/64	495/252	615/290
#Non-essential	348	336	253	593	305	407
#Overlapping genes	95		68			

^*^Persistence was computed for each essential and conditional essential genes over the 58 *Pseudomonas* genomes.
