# Supplementary material for: Comparison of 432 Pseudomonas strains through integration of genomic, functional, metabolic and expression data
Source: Sci Rep. 2016 Dec 6;6:38699. doi: 10.1038/srep38699 (PMC5138606; doi:10.1038/srep38699)
Supplement: Supplementary Information [file srep38699-s2.pdf]

Comparison of 432 Pseudomonas strains through integration of genomic, functional, metabolic and expression data

Jasper J Koehorst[1,+], Jesse CJ van Dam[1,+], Ruben GA van Heck[1], Edoardo Saccenti[1], Vitor AP Martins dos Santos[1,2], Maria Suarez-Diez[1,=*], and Peter J Schaap[1,=]

[1]Wageningen University, Laboratory of Systems and Synthetic Biology, Wageningen, 6708 WE, The Netherlands [2]LifeGlimmer GmbH, Berlin, Germany [*]maria.suarezdiez@wur.nl

[+]these authors contributed equally to this work [=]these authors contributed equally to this work

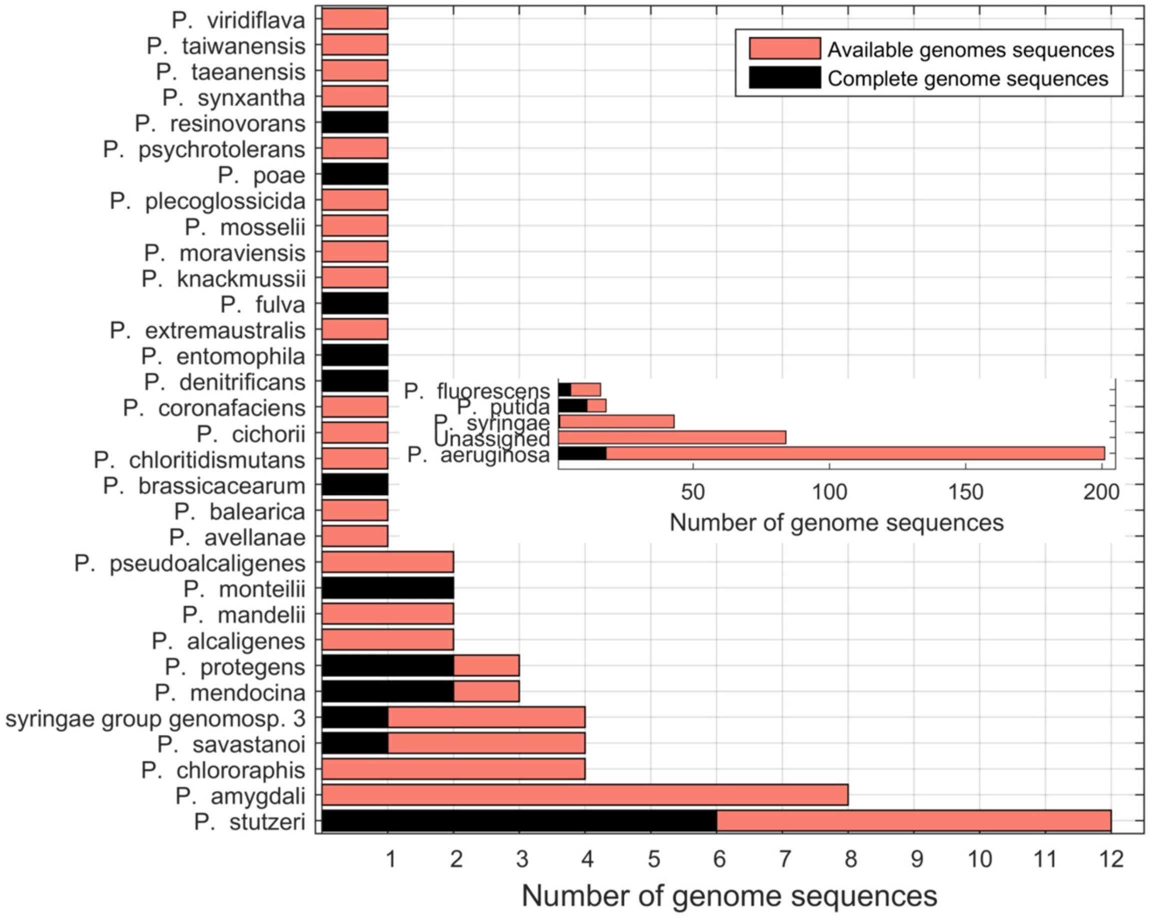

Supplementary Figure 1:

Overview of available Pseudomonas genome sequences analyzed in this study arranged per species.

Supplementary Table 2:

Comparison between the de-novo annotation and the original annotation. *Date* refers to the deposition date of the genome sequence in the NCBI database as retrieved from https://www.ncbi.nlm.nih.gov/. #Genes, #Domains, #GO and #ECs indicate the difference between the total number of genes, protein domains, GO terms and EC numbers between the *de-novo* and the original annotation, respectively.

Organism	Date	#Genes	#Domains	#Go	#ECs
**Pseudomonas_aeruginosa_PAO1**	16/05/00	109	4	17	2
**Pseudomonas_putida_KT2440**	08/04/02	244	14	94	4
**Pseudomonas_syringae_pv__tomato_str__DC3000**	25/03/03	245	2	38	1
**Pseudomonas_putida_ND6**	20/05/03	-682	-2	31	1
**Pseudomonas_syringae_pv__phaseolicola_1448A**	04/03/04	407	4	50	2
**Pseudomonas_syringae_pv__syringae_B728a**	04/05/05	118	-1	-2	1
**Pseudomonas_protegens_Pf_5**	13/05/05	168	24	1	0
**Pseudomonas_fluorescens_Pf0_1**	08/08/05	17	1	-25	0
**Pseudomonas_fluorescens_SBW25**	09/01/06	109	2	20	0
**Pseudomonas_entomophila_L48**	27/01/06	-61	6	-7	2
**Pseudomonas_stutzeri_A1501**	03/04/06	49	5	41	-1
**Pseudomonas_aeruginosa_UCBPP_PA14**	18/08/06	16	-3	0	0
**Pseudomonas_aeruginosa_PA7**	06/09/06	-255	-1	0	0
**Pseudomonas_mendocina_ymp**	17/04/07	39	-1	11	0
**Pseudomonas_putida_F1**	25/05/07	45	-1	-7	1
**Pseudomonas_putida_GB_1**	24/01/08	37	1	-6	0
**Pseudomonas_putida_W619**	21/02/08	55	4	-7	0
**Pseudomonas_aeruginosa_LESB58**	02/09/08	134	4	34	0
**Pseudomonas_putida_BIRD_1**	18/10/10	191	5	46	0
**Pseudomonas_mendocina_NK_01**	01/01/11	50	2	28	2
**Pseudomonas_aeruginosa_M18**	18/01/11	87	4	-6	2
**Pseudomonas_brassicacearum_subsp...NFM421**	11/03/11	-39	3	-3	0
**Pseudomonas_stutzeri_DSM_4166**	29/03/11	33	3	28	2
**Pseudomonas_fulva_12_X**	29/04/11	2	-13	0	0
**Pseudomonas_stutzeri_ATCC_17588___LMG_11199**	22/06/11	-20	2	28	0
**Pseudomonas_putida_S16**	28/06/11	249	0	41	0
**Pseudomonas_fluorescens_A506**	09/09/11	102	2	11	0
**Pseudomonas_stutzeri_RCH2**	05/10/11	39	0	6	0
**Pseudomonas_aeruginosa_DK2**	22/11/11	-12	2	-2	2

**Pseudomonas_fluorescens_F113**	22/11/11	183	4	12	0
**Pseudomonas_protegens_CHA0**	08/12/11	26	7	37	1
**Pseudomonas_fluorescens_R124**	05/03/12	0	0	0	0
**Pseudomonas_aeruginosa_NCGM2_S1**	10/04/12	-42	3	50	1
**Pseudomonas_stutzeri_CCUG_29243**	11/06/12	56	0	-9	0
**Pseudomonas_putida_DOT_T1E**	04/07/12	29	47	0	0
**Pseudomonas_stutzeri_DSM_10701**	10/07/12	23	0	13	0
**Pseudomonas_putida_HB3267**	23/07/12	39	0	-5	1
**Pseudomonas_sp__UW4**	17/09/12	70	-5	0	0
**Pseudomonas_sp__VLB120**	26/11/12	53	4	1	0
**Pseudomonas_aeruginosa_PA1**	25/12/12	182	5	106	0
**Pseudomonas_aeruginosa_PA1R**	25/12/12	179	5	107	0
**Pseudomonas_poae_RE_1_1_14**	11/01/13	2	0	-22	0
**Pseudomonas_denitrificans_ATCC_13867**	15/02/13	5	3	-2	2
**Pseudomonas_aeruginosa_B136_33**	09/04/13	-10	-2	-19	0
**Pseudomonas_putida_H8234**	06/05/13	33	5	31	0
**Pseudomonas_resinovorans_NBRC_106553**	04/06/13	-105	1	-22	0
**Pseudomonas_putida_NBRC_14164**	14/06/13	-14	0	-1	0
**Pseudomonas_aeruginosa_RP73**	17/06/13	42	1	16	1
**Pseudomonas_chloritidismutans_AW_1**	22/07/13	0	4	31	1
**Pseudomonas_aeruginosa_PAO581**	14/08/13	36	2	2	1
**Pseudomonas_aeruginosa_c7447m**	05/09/13	32	-2	3	0
**Pseudomonas_aeruginosa_PAO1_VE13**	22/10/13	11	1	4	0
**Pseudomonas_sp__TKP**	14/11/13	-50	0	-23	0
**Pseudomonas_aeruginosa_MTB_1**	27/11/13	-1	-21	1	0
**Pseudomonas_aeruginosa_SCV20265**	02/12/13	-109	3	39	0
**Pseudomonas_aeruginosa_LES431**	03/12/13	42	4	5	0
**Pseudomonas_monteilii_SB3078**	11/12/13	23	3	12	0
**Pseudomonas_monteilii_SB3101**	11/12/13	24	2	11	1

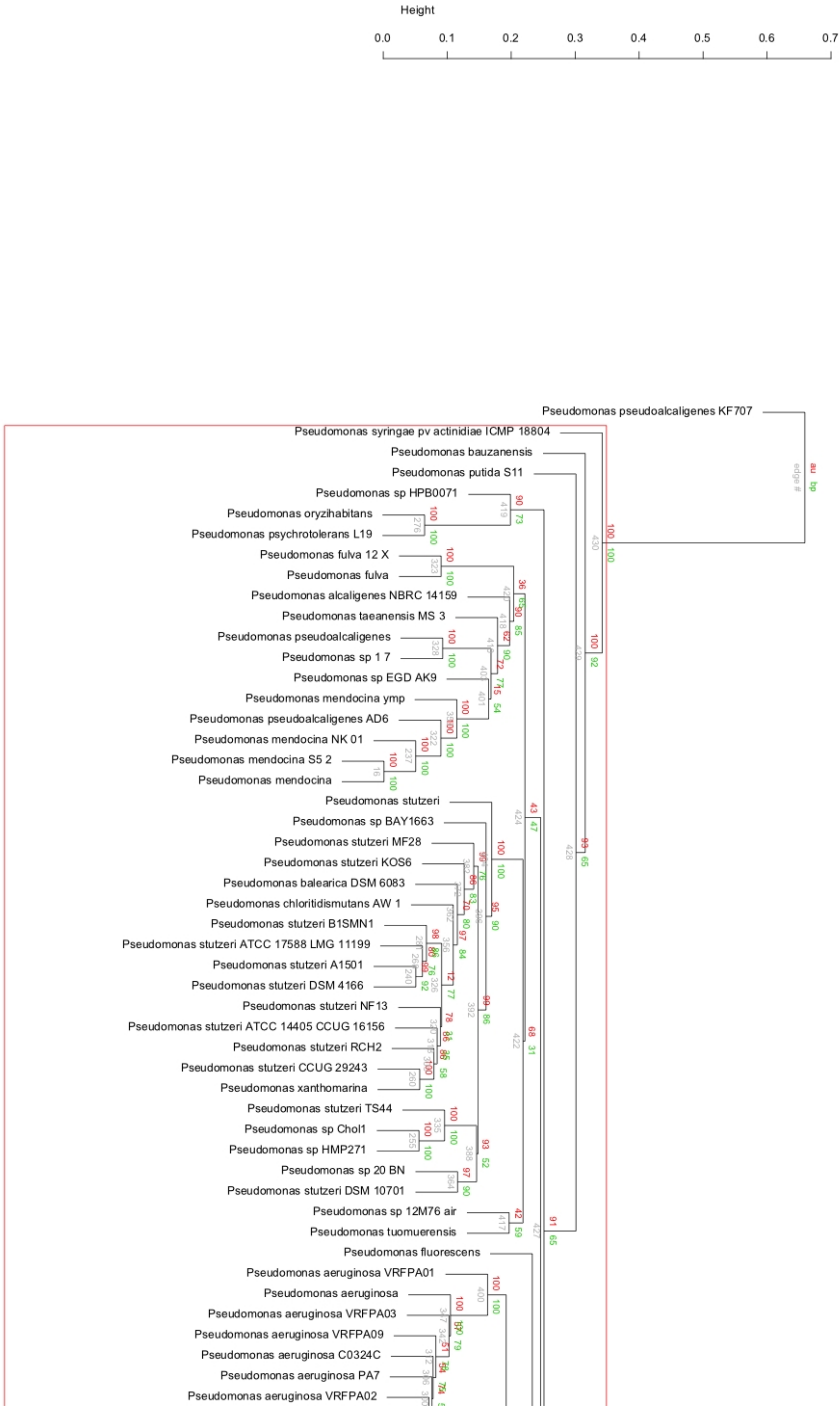
Height
0.0
0.1
0.2
0.3
0.4
0.5
0.6
0.7
au
bp
edge #
Pseudomonas pseudoalcaligenes KF707
Pseudomonas syringae pv actinidiae ICMP 18804
Pseudomonas bauzanensis
Pseudomonas putida S11
Pseudomonas sp HPB0071
Pseudomonas oryzihabitans
Pseudomonas psychrotolerans L19
Pseudomonas fulva 12 X
Pseudomonas fulva
Pseudomonas alcaligenes NBRC 14159
Pseudomonas taeanensis MS 3
Pseudomonas pseudoalcaligenes
Pseudomonas sp 1 7
Pseudomonas sp EGD AK9
Pseudomonas mendocina ymp
Pseudomonas pseudoalcaligenes AD6
Pseudomonas mendocina NK 01
Pseudomonas mendocina S5 2
Pseudomonas mendocina
Pseudomonas stutzeri
Pseudomonas sp BAY1663
Pseudomonas stutzeri MF28
Pseudomonas stutzeri KOS6
Pseudomonas balearica DSM 6083
Pseudomonas chloritidismutans AW 1
Pseudomonas stutzeri B1SMN1
Pseudomonas stutzeri ATCC 17588 LMG 11199
Pseudomonas stutzeri A1501
Pseudomonas stutzeri DSM 4166
Pseudomonas stutzeri NF13
Pseudomonas stutzeri ATCC 14405 CCUG 16156
Pseudomonas stutzeri RCH2
Pseudomonas stutzeri CCUG 29243
Pseudomonas xanthomarina
Pseudomonas stutzeri TS44
Pseudomonas sp Chol1
Pseudomonas sp HMP271
Pseudomonas sp 20 BN
Pseudomonas stutzeri DSM 10701
Pseudomonas sp 12M76 air
Pseudomonas tuomuerensis
Pseudomonas fluorescens
Pseudomonas aeruginosa VRFPA01
Pseudomonas aeruginosa
Pseudomonas aeruginosa VRFPA03
Pseudomonas aeruginosa VRFPA09
Pseudomonas aeruginosa C0324C
Pseudomonas aeruginosa PA7
Pseudomonas aeruginosa VRFPA02

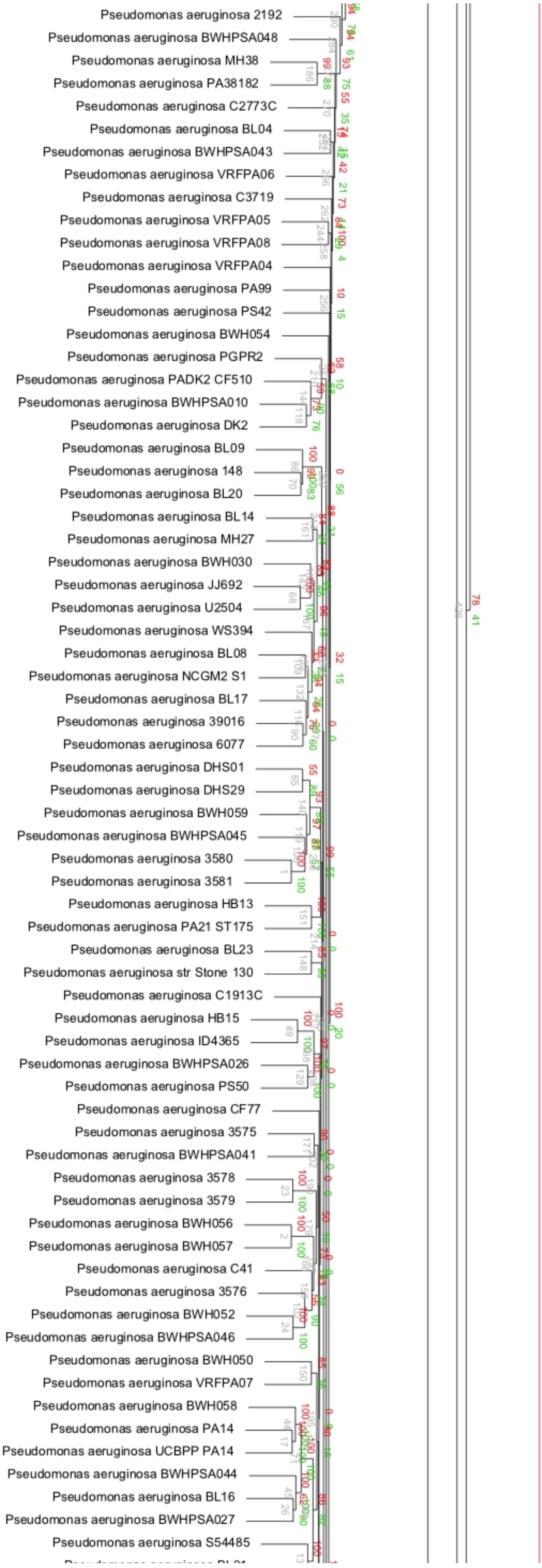

Pseudomonas aeruginosa 2192
Pseudomonas aeruginosa BWHPSA048
Pseudomonas aeruginosa MH38
Pseudomonas aeruginosa PA38182
Pseudomonas aeruginosa C2773C
Pseudomonas aeruginosa BL04
Pseudomonas aeruginosa BWHPSA043
Pseudomonas aeruginosa VRFPA06
Pseudomonas aeruginosa C3719
Pseudomonas aeruginosa VRFPA05
Pseudomonas aeruginosa VRFPA08
Pseudomonas aeruginosa VRFPA04
Pseudomonas aeruginosa PA99
Pseudomonas aeruginosa PS42
Pseudomonas aeruginosa BWH054
Pseudomonas aeruginosa PGPR2
Pseudomonas aeruginosa PADK2 CF510
Pseudomonas aeruginosa BWHPSA010
Pseudomonas aeruginosa DK2
Pseudomonas aeruginosa BL09
Pseudomonas aeruginosa 148
Pseudomonas aeruginosa BL20
Pseudomonas aeruginosa BL14
Pseudomonas aeruginosa MH27
Pseudomonas aeruginosa BWH030
Pseudomonas aeruginosa JJ692
Pseudomonas aeruginosa U2504
Pseudomonas aeruginosa WS394
Pseudomonas aeruginosa BL08
Pseudomonas aeruginosa NCGM2 S1
Pseudomonas aeruginosa BL17
Pseudomonas aeruginosa 39016
Pseudomonas aeruginosa 6077
Pseudomonas aeruginosa DHS01
Pseudomonas aeruginosa DHS29
Pseudomonas aeruginosa BWH059
Pseudomonas aeruginosa BWHPSA045
Pseudomonas aeruginosa 3580
Pseudomonas aeruginosa 3581
Pseudomonas aeruginosa HB13
Pseudomonas aeruginosa PA21 ST175
Pseudomonas aeruginosa BL23
Pseudomonas aeruginosa str Stone 130
Pseudomonas aeruginosa C1913C
Pseudomonas aeruginosa HB15
Pseudomonas aeruginosa ID4365
Pseudomonas aeruginosa BWHPSA026
Pseudomonas aeruginosa PS50
Pseudomonas aeruginosa CF77
Pseudomonas aeruginosa 3575
Pseudomonas aeruginosa BWHPSA041
Pseudomonas aeruginosa 3578
Pseudomonas aeruginosa 3579
Pseudomonas aeruginosa BWH056
Pseudomonas aeruginosa BWH057
Pseudomonas aeruginosa C41
Pseudomonas aeruginosa 3576
Pseudomonas aeruginosa BWH052
Pseudomonas aeruginosa BWHPSA046
Pseudomonas aeruginosa BWH050
Pseudomonas aeruginosa VRFPA07
Pseudomonas aeruginosa BWH058
Pseudomonas aeruginosa PA14
Pseudomonas aeruginosa UCBPP PA14
Pseudomonas aeruginosa BWHPSA044
Pseudomonas aeruginosa BL16
Pseudomonas aeruginosa BWHPSA027
Pseudomonas aeruginosa S54485

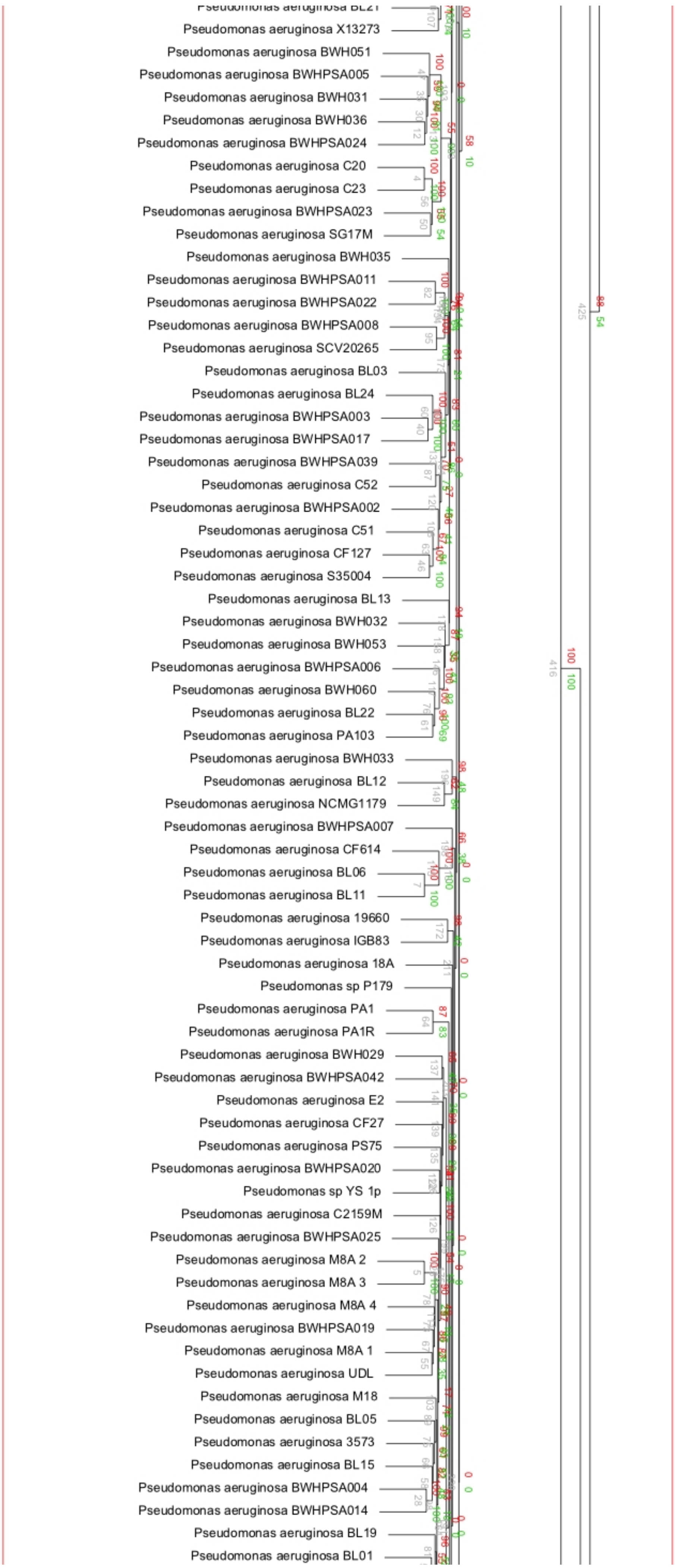
Pseudomonas aeruginosa X13273
Pseudomonas aeruginosa BWH051
Pseudomonas aeruginosa BWHPSA005
Pseudomonas aeruginosa BWH031
Pseudomonas aeruginosa BWH036
Pseudomonas aeruginosa BWHPSA024
Pseudomonas aeruginosa C20
Pseudomonas aeruginosa C23
Pseudomonas aeruginosa BWHPSA023
Pseudomonas aeruginosa SG17M
Pseudomonas aeruginosa BWH035
Pseudomonas aeruginosa BWHPSA011
Pseudomonas aeruginosa BWHPSA022
Pseudomonas aeruginosa BWHPSA008
Pseudomonas aeruginosa SCV20265
Pseudomonas aeruginosa BL03
Pseudomonas aeruginosa BL24
Pseudomonas aeruginosa BWHPSA003
Pseudomonas aeruginosa BWHPSA017
Pseudomonas aeruginosa BWHPSA039
Pseudomonas aeruginosa C52
Pseudomonas aeruginosa BWHPSA002
Pseudomonas aeruginosa C51
Pseudomonas aeruginosa CF127
Pseudomonas aeruginosa S35004
Pseudomonas aeruginosa BL13
Pseudomonas aeruginosa BWH032
Pseudomonas aeruginosa BWH053
Pseudomonas aeruginosa BWHPSA006
Pseudomonas aeruginosa BWH060
Pseudomonas aeruginosa BL22
Pseudomonas aeruginosa PA103
Pseudomonas aeruginosa BWH033
Pseudomonas aeruginosa BL12
Pseudomonas aeruginosa NCMG1179
Pseudomonas aeruginosa BWHPSA007
Pseudomonas aeruginosa CF614
Pseudomonas aeruginosa BL06
Pseudomonas aeruginosa BL11
Pseudomonas aeruginosa 19660
Pseudomonas aeruginosa IGB83
Pseudomonas aeruginosa 18A
Pseudomonas sp P179
Pseudomonas aeruginosa PA1
Pseudomonas aeruginosa PA1R
Pseudomonas aeruginosa BWH029
Pseudomonas aeruginosa BWHPSA042
Pseudomonas aeruginosa E2
Pseudomonas aeruginosa CF27
Pseudomonas aeruginosa PS75
Pseudomonas aeruginosa BWHPSA020
Pseudomonas sp YS 1p
Pseudomonas aeruginosa C2159M
Pseudomonas aeruginosa BWHPSA025
Pseudomonas aeruginosa M8A 2
Pseudomonas aeruginosa M8A 3
Pseudomonas aeruginosa M8A 4
Pseudomonas aeruginosa BWHPSA019
Pseudomonas aeruginosa M8A 1
Pseudomonas aeruginosa UDL
Pseudomonas aeruginosa M18
Pseudomonas aeruginosa BL05
Pseudomonas aeruginosa 3573
Pseudomonas aeruginosa BL15
Pseudomonas aeruginosa BWHPSA004
Pseudomonas aeruginosa BWHPSA014
Pseudomonas aeruginosa BL19
Pseudomonas aeruginosa BL01

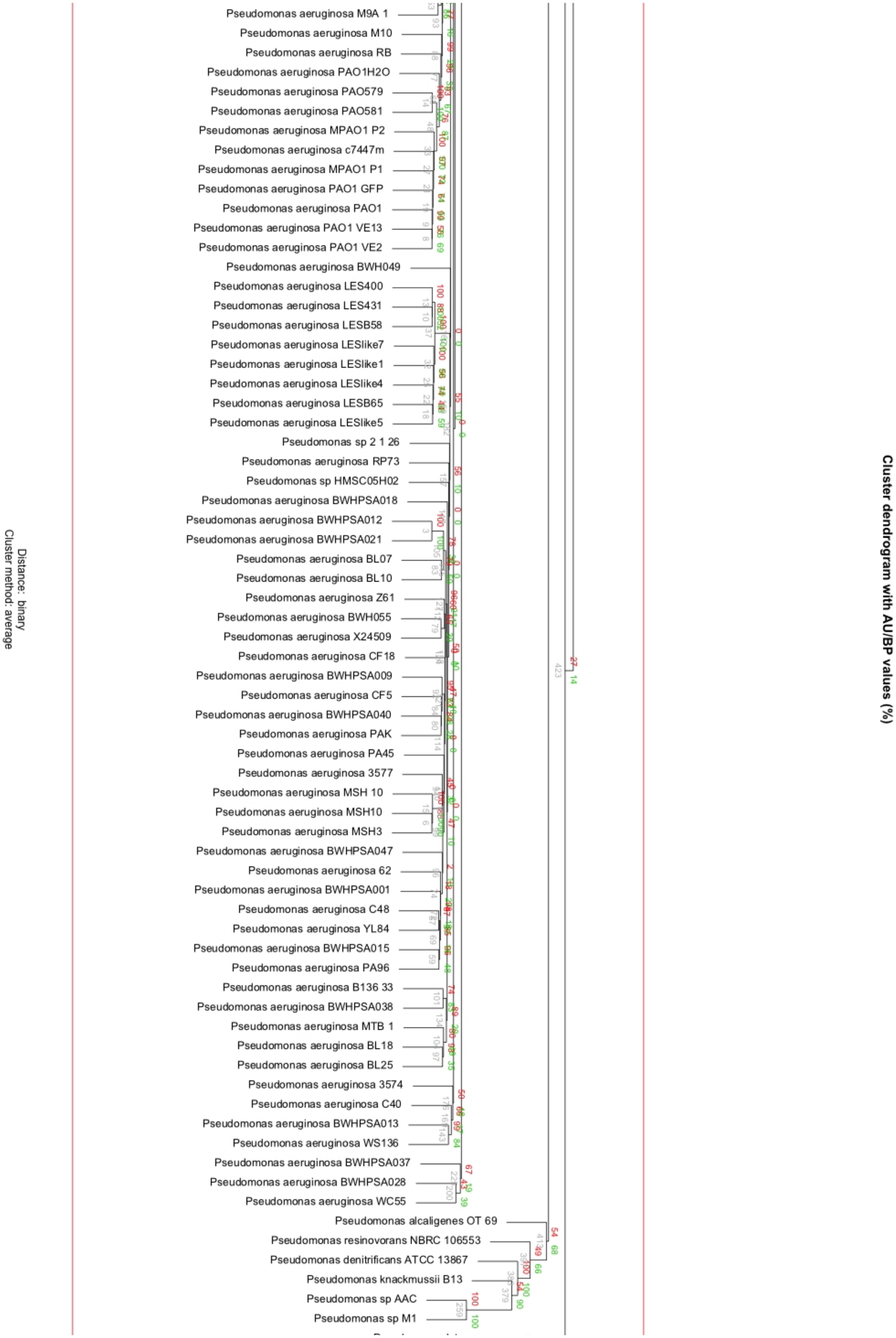

Cluster dendrogram with AU/BP values (%)
Pseudomonas aeruginosa M9A 1
Pseudomonas aeruginosa M10
Pseudomonas aeruginosa RB
Pseudomonas aeruginosa PAO1H2O
Pseudomonas aeruginosa PAO579
Pseudomonas aeruginosa PAO581
Pseudomonas aeruginosa MPAO1 P2
Pseudomonas aeruginosa c7447m
Pseudomonas aeruginosa MPAO1 P1
Pseudomonas aeruginosa PAO1 GFP
Pseudomonas aeruginosa PAO1
Pseudomonas aeruginosa PAO1 VE13
Pseudomonas aeruginosa PAO1 VE2
Pseudomonas aeruginosa BWH049
Pseudomonas aeruginosa LES400
Pseudomonas aeruginosa LES431
Pseudomonas aeruginosa LESB58
Pseudomonas aeruginosa LESlike7
Pseudomonas aeruginosa LESlike1
Pseudomonas aeruginosa LESlike4
Pseudomonas aeruginosa LESB65
Pseudomonas aeruginosa LESlike5
Pseudomonas sp 2 1 26
Pseudomonas aeruginosa RP73
Pseudomonas sp HMSC05H02
Pseudomonas aeruginosa BWHPSA018
Pseudomonas aeruginosa BWHPSA012
Pseudomonas aeruginosa BWHPSA021
Pseudomonas aeruginosa BL07
Pseudomonas aeruginosa BL10
Pseudomonas aeruginosa Z61
Pseudomonas aeruginosa BWH055
Pseudomonas aeruginosa X24509
Pseudomonas aeruginosa CF18
Pseudomonas aeruginosa BWHPSA009
Pseudomonas aeruginosa CF5
Pseudomonas aeruginosa BWHPSA040
Pseudomonas aeruginosa PAK
Pseudomonas aeruginosa PA45
Pseudomonas aeruginosa 3577
Pseudomonas aeruginosa MSH 10
Pseudomonas aeruginosa MSH10
Pseudomonas aeruginosa MSH3
Pseudomonas aeruginosa BWHPSA047
Pseudomonas aeruginosa 62
Pseudomonas aeruginosa BWHPSA001
Pseudomonas aeruginosa C48
Pseudomonas aeruginosa YL84
Pseudomonas aeruginosa BWHPSA015
Pseudomonas aeruginosa PA96
Pseudomonas aeruginosa B136 33
Pseudomonas aeruginosa BWHPSA038
Pseudomonas aeruginosa MTB 1
Pseudomonas aeruginosa BL18
Pseudomonas aeruginosa BL25
Pseudomonas aeruginosa 3574
Pseudomonas aeruginosa C40
Pseudomonas aeruginosa BWHPSA013
Pseudomonas aeruginosa WS136
Pseudomonas aeruginosa BWHPSA037
Pseudomonas aeruginosa BWHPSA028
Pseudomonas aeruginosa WC55
Pseudomonas alcaligenes OT 69
Pseudomonas resinovorans NBRC 106553
Pseudomonas denitrificans ATCC 13867
Pseudomonas knackmussii B13
Pseudomonas sp AAC
Pseudomonas sp M1
Distance: binary
Cluster method: average

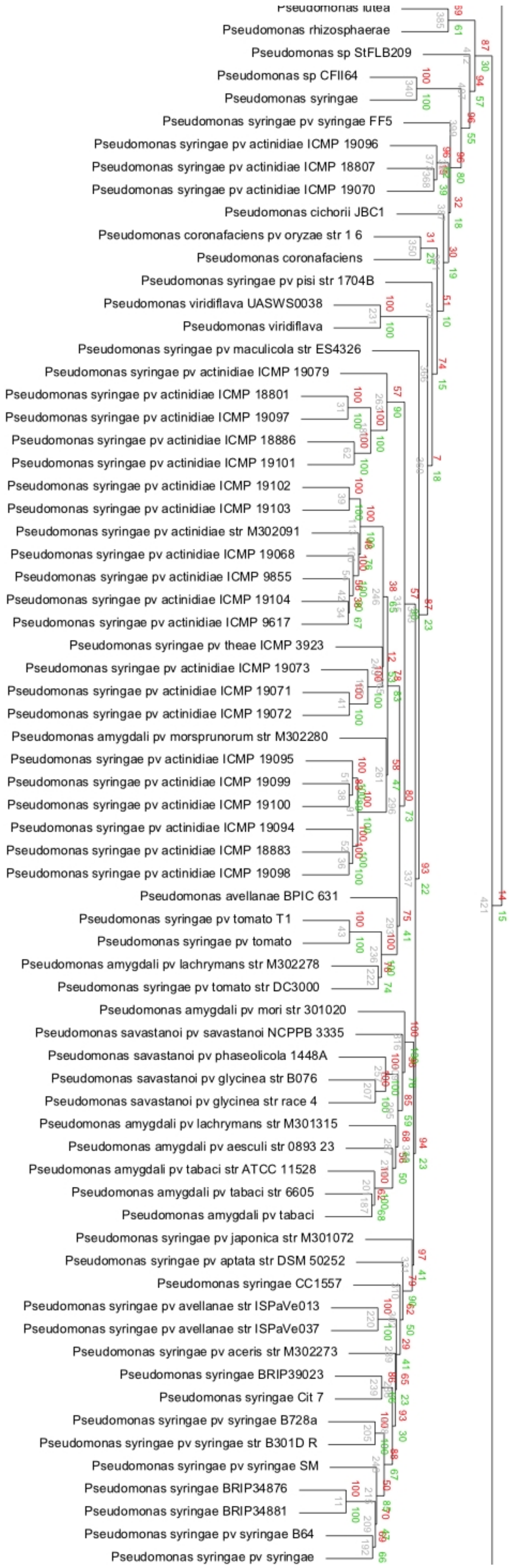
Pseudomonas lutea
Pseudomonas rhizosphaerae
Pseudomonas sp StFLB209
Pseudomonas sp CFII64
Pseudomonas syringae
Pseudomonas syringae pv syringae FF5
Pseudomonas syringae pv actinidiae ICMP 19096
Pseudomonas syringae pv actinidiae ICMP 18807
Pseudomonas syringae pv actinidiae ICMP 19070
Pseudomonas cichorii JBC1
Pseudomonas coronafaciens pv oryzae str 1 6
Pseudomonas coronafaciens
Pseudomonas syringae pv pisi str 1704B
Pseudomonas viridiflava UASWS0038
Pseudomonas viridiflava
Pseudomonas syringae pv maculicola str ES4326
Pseudomonas syringae pv actinidiae ICMP 19079
Pseudomonas syringae pv actinidiae ICMP 18801
Pseudomonas syringae pv actinidiae ICMP 19097
Pseudomonas syringae pv actinidiae ICMP 18886
Pseudomonas syringae pv actinidiae ICMP 19101
Pseudomonas syringae pv actinidiae ICMP 19102
Pseudomonas syringae pv actinidiae ICMP 19103
Pseudomonas syringae pv actinidiae str M302091
Pseudomonas syringae pv actinidiae ICMP 19068
Pseudomonas syringae pv actinidiae ICMP 9855
Pseudomonas syringae pv actinidiae ICMP 19104
Pseudomonas syringae pv actinidiae ICMP 9617
Pseudomonas syringae pv theae ICMP 3923
Pseudomonas syringae pv actinidiae ICMP 19073
Pseudomonas syringae pv actinidiae ICMP 19071
Pseudomonas syringae pv actinidiae ICMP 19072
Pseudomonas amygdali pv morsprunorum str M302280
Pseudomonas syringae pv actinidiae ICMP 19095
Pseudomonas syringae pv actinidiae ICMP 19099
Pseudomonas syringae pv actinidiae ICMP 19100
Pseudomonas syringae pv actinidiae ICMP 19094
Pseudomonas syringae pv actinidiae ICMP 18883
Pseudomonas syringae pv actinidiae ICMP 19098
Pseudomonas avellanae BPIC 631
Pseudomonas syringae pv tomato T1
Pseudomonas syringae pv tomato
Pseudomonas amygdali pv lachrymans str M302278
Pseudomonas syringae pv tomato str DC3000
Pseudomonas amygdali pv mori str 301020
Pseudomonas savastanoi pv savastanoi NCPPB 3335
Pseudomonas savastanoi pv phaseolicola 1448A
Pseudomonas savastanoi pv glycinea str B076
Pseudomonas savastanoi pv glycinea str race 4
Pseudomonas amygdali pv lachrymans str M301315
Pseudomonas amygdali pv aesculi str 0893 23
Pseudomonas amygdali pv tabaci str ATCC 11528
Pseudomonas amygdali pv tabaci str 6605
Pseudomonas amygdali pv tabaci
Pseudomonas syringae pv japonica str M301072
Pseudomonas syringae pv aptata str DSM 50252
Pseudomonas syringae CC1557
Pseudomonas syringae pv avellanae str ISPaVe013
Pseudomonas syringae pv avellanae str ISPaVe037
Pseudomonas syringae pv aceris str M302273
Pseudomonas syringae BRIP39023
Pseudomonas syringae Cit 7
Pseudomonas syringae pv syringae B728a
Pseudomonas syringae pv syringae str B301D R
Pseudomonas syringae pv syringae SM
Pseudomonas syringae BRIP34876
Pseudomonas syringae BRIP34881
Pseudomonas syringae pv syringae B64
Pseudomonas syringae pv syringae

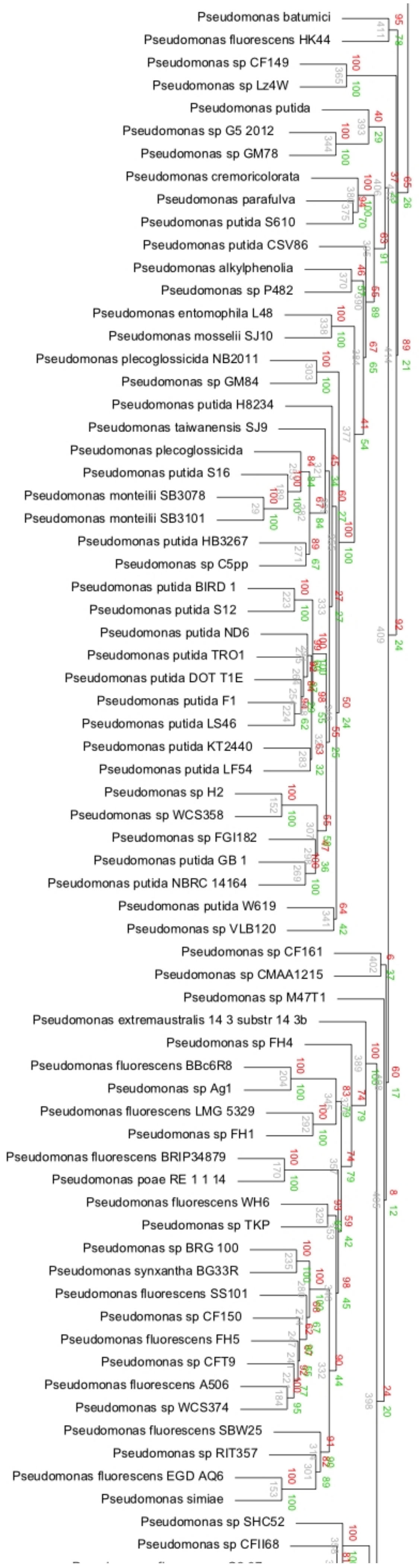
Pseudomonas batumici
Pseudomonas fluorescens HK44
Pseudomonas sp CF149
Pseudomonas sp Lz4W
Pseudomonas putida
Pseudomonas sp G5 2012
Pseudomonas sp GM78
Pseudomonas cremoricolorata
Pseudomonas parafulva
Pseudomonas putida S610
Pseudomonas putida CSV86
Pseudomonas alkylphenolia
Pseudomonas sp P482
Pseudomonas entomophila L48
Pseudomonas mosselii SJ10
Pseudomonas plecoglossicida NB2011
Pseudomonas sp GM84
Pseudomonas putida H8234
Pseudomonas taiwanensis SJ9
Pseudomonas plecoglossicida
Pseudomonas putida S16
Pseudomonas monteilii SB3078
Pseudomonas monteilii SB3101
Pseudomonas putida HB3267
Pseudomonas sp C5pp
Pseudomonas putida BIRD 1
Pseudomonas putida S12
Pseudomonas putida ND6
Pseudomonas putida TRO1
Pseudomonas putida DOT T1E
Pseudomonas putida F1
Pseudomonas putida LS46
Pseudomonas putida KT2440
Pseudomonas putida LF54
Pseudomonas sp H2
Pseudomonas sp WCS358
Pseudomonas sp FGI182
Pseudomonas putida GB 1
Pseudomonas putida NBRC 14164
Pseudomonas putida W619
Pseudomonas sp VLB120
Pseudomonas sp CF161
Pseudomonas sp CMAA1215
Pseudomonas sp M47T1
Pseudomonas extremaustralis 14 3 substr 14 3b
Pseudomonas sp FH4
Pseudomonas fluorescens BBc6R8
Pseudomonas sp Ag1
Pseudomonas fluorescens LMG 5329
Pseudomonas sp FH1
Pseudomonas fluorescens BRIP34879
Pseudomonas poae RE 1 1 14
Pseudomonas fluorescens WH6
Pseudomonas sp TKP
Pseudomonas sp BRG 100
Pseudomonas synxantha BG33R
Pseudomonas fluorescens SS101
Pseudomonas sp CF150
Pseudomonas fluorescens FH5
Pseudomonas sp CFT9
Pseudomonas fluorescens A506
Pseudomonas sp WCS374
Pseudomonas fluorescens SBW25
Pseudomonas sp RIT357
Pseudomonas fluorescens EGD AQ6
Pseudomonas simiae
Pseudomonas sp SHC52
Pseudomonas sp CFII68

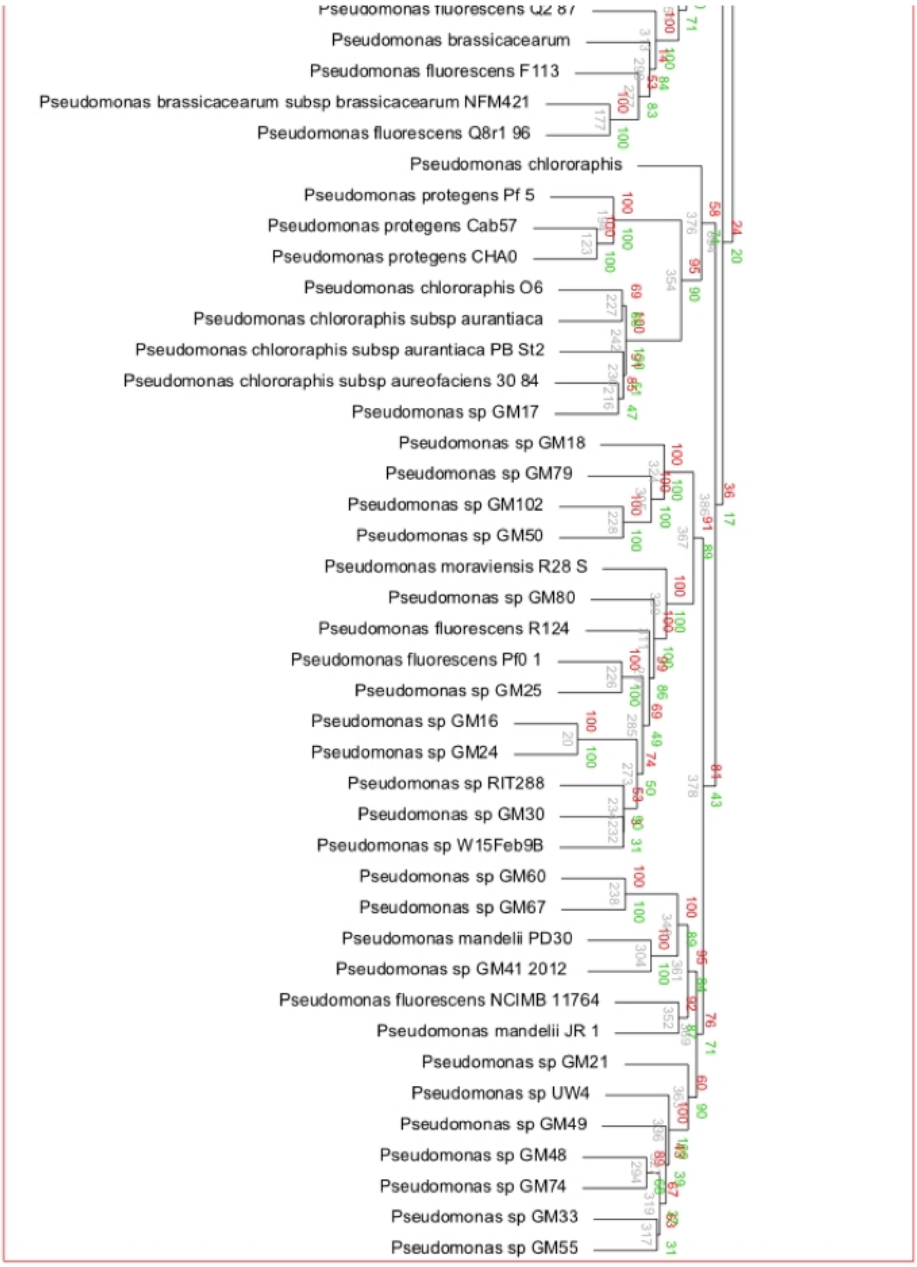

Supplementary Figure 3:
Pan-genome tree for 432 *Pseudomonas* genomes based on protein domains.
A binarized matrix indicating presence-absence of protein domains for each considered genome was obtained. The hierarchical tree was constructed using as input a Manhattan distance matrix derived from this matrix

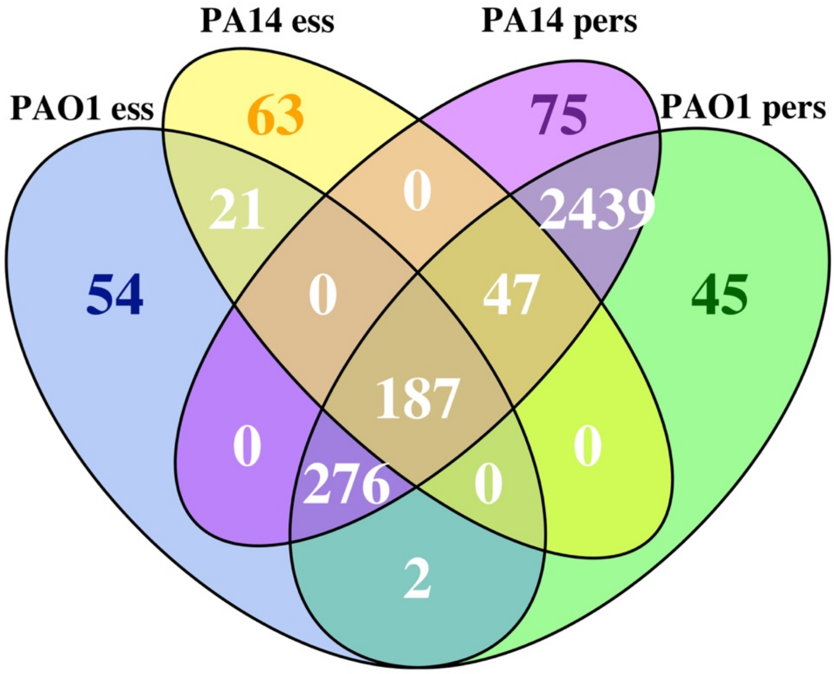

Supplementary Figure 4:
A comparison of gene persistence and essentiality for *P. aeruginosa* PAO1 and *P. aeruginosa* PA14. PA14 ess: refers to genes found to be essential for growth of *P. aeruginosa* PA14 on LB (see Liberati *et al* 2006 PNAS); **PAO1 ess**: genes found to be essential for growth of *P. aeruginosa* on either LB, minimal medium with pyruvate addition or sputum agar (see Lee *et al* 2015 PNAS). **PA14 pers** and **PAO1 pers** indicate genes that are present P. aeruginosa PA14 and PAO1 respectively and have a high (>0.95) persistence in the Pseudomonas genus.

Variability of gene expression profiles and its association to essentiality and persistence for *P. aeruginosa* PA14 using RNAseq data

An RNAseq dataset corresponding to *P. aeruginosa* PA14 grown under 14 different environmental conditions (different growth temperatures, stages in the growth curve, osmolarity, phosphate and iron concentrations, oxygen availability, surface attachment or within biofilms) was used to asses gene expression variability in this strain (Dötsch *et al.* 2015). On the basis of differential expression analysis each gene was assigned a score, *FCS*, for transcriptional variation. Persistent genes tend to show significantly lower degree of variation in expression level than those with lower persistence (p-value <0.01); this holds true also for essential genes (Fig. S1)

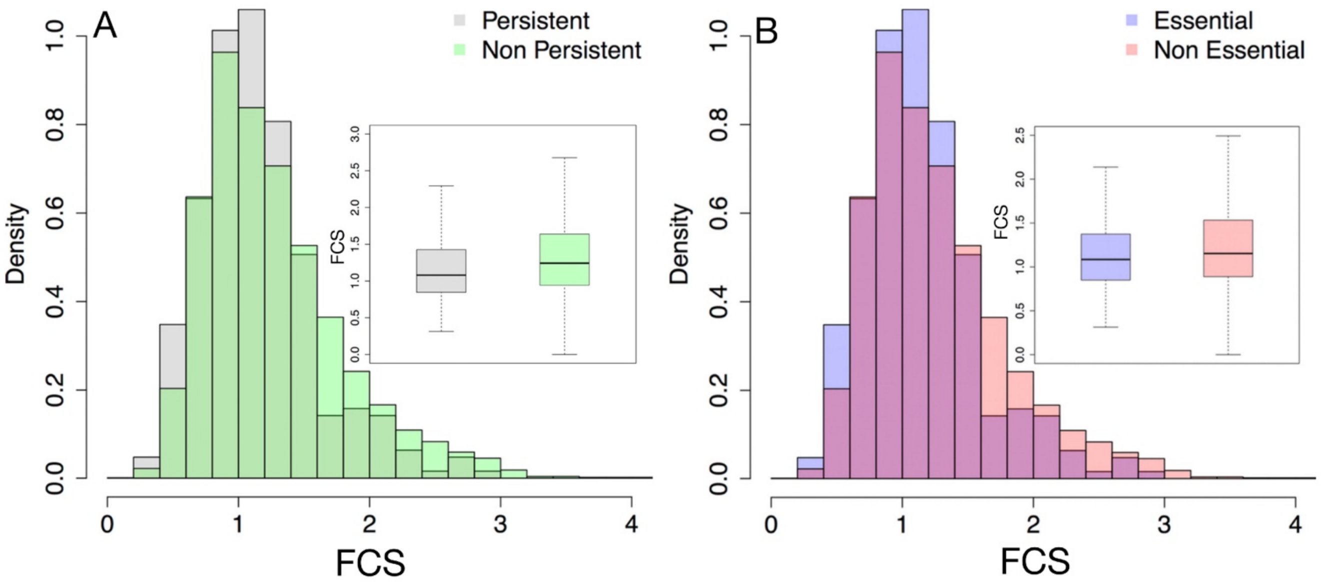

Figure S1: Variability of gene expression levels and its association with persistence and essentiality fro P. aeruginosa *PA14 usin RNASeq A) Distribution of* FCS *index for (non) persistent genes (genes with persistence lower or higher than 0.95, respectively). The box blot gives the average value of* FCS *for the two groups. The difference between the mean values is significant* (p-*val<0.01). B) Distribution of* FCS i*ndex for essential and non-essential genes with gene essentiality derived experimentally (Liberati* et al. *2006). The box blot gives the average value of FCS for the two groups. The difference between the mean values is significant* (p-*val<0.01)*

FCS Computation

Number of RNA-seq reads mapped to the genome of *P. aeruginosa* PA14 grown under 14 different were retrieved from the GEO database (accession number: GSE55197 (Dötsch *et al.* 2015). The data corresponds to the environmental growth conditions within biofilms, at various temperatures, osmolarities and phosphate concentrations, under anaerobic conditions, attached to a surface and conditions encountered within the eukaryotic host. For every gene we calculated the $\log_2$ fold change of its expression values when considering all possible pairwise comparison of conditions. These values were aggregated in an overall fold change score, *FCS*, by averaging their absolute values. Missing values for fold change in individual comparisons arising from genes with very low counts in some conditions were imputed to 0 or ±4, according to the significance of the differential expression (fdr<0.05 selected as a threshold).

Supplementary Data 6: Models and the code used to analyze the six metabolic models.
